# New Method with Dahlias

**Published:** 1886-07

**Authors:** 


### A NEW METHOD WITH DAHLIAS.

A new method in Dahlia cultivation has been successfully practiced in France. It consists in pegging down the stems of the plants as they grow ; by so doing the foliage presents a horizontal surface—a carpet of green—through which rise the flower stems bearing the blooms.

In planting a bed for Dahlias to be trained in this manner, set the plants with a slight inclination, in order to favor laying down the stems afterwards. The stems are to be fastened down as they grow, and so arranged as in time to cover all the soil. Wooden pegs are used for fastening, the same as in pegging down Verbenas, Petunias, and other plants.

The only care necessary is to direct the flower-stems to an upright position.

Plants of strong-growing varieties will cover a space of a square yard or more. All the stems and branches should be preserved and allowed to grow their full length.

The pegs which are used to keep the stems in place at the commencement can afterwards be pulled out and used again, thus economizing them.

According to the writer in the *Revue Horticole*, from which these notes are taken, this mode of culture will adapt itself to a great number of ornamental combinations. Borders can be formed of single-colored flowers, or, on the contrary, the colors can be varied, and every other plant can be introduced according to some prepared design.

In planting large grounds these ideas are well worthy of being put into practice, and the skillful gardener will readily perceive the advantages offered.—*Vick's Magazine.*

---

**The best method of removing foreign bodies from the ear. Says** Dr. Jonathan Hutchinson in the *Brit. Med. Jour.* of April 10, 1886, is so simple and efficient that it almost supersedes the need of knowledge. It is the use of a silver wireloop, instead of either forceps or scoop. I have never, since I was a student, used either of the latter instruments ; and, for the purpose of extracting hard bodies from the ear, I hold that they are most dangerous. With a flexible silver wire-loop, or, if need be, with two placed at right angles, I have repeatedly succeeded when all other means had failed. Thus, not only is the loop quite devoid of danger, but it is both more easy of use and far more efficient than any other method. It is impossible that it can injure the membrana tympani, or the walls of the canal.

The method of proceedure is, after having put the patient under anæsthetics, is to introduce the loop gently into the ear, and turn it about until it is believed to have got behind the foreign body. This it will often do at once ; but sometimes a little patience is necessary. In one instance, I took out a piece of heavy lead in this way with very little trouble, using two loops at right angles with each other. The simplicity, safety, and effeciency of the method make it desirable that it should be better known.

